# Measurement of Calprotectin and PTH in the Amniotic Fluid of Early Second Trimester Pregnancies and Their Impact on Fetuses with Growth Disorders: Are Their Levels Related to Oxidative Stress?

**DOI:** 10.3390/jcm13030855

**Published:** 2024-02-01

**Authors:** George Maroudias, Dionysios Vrachnis, Alexandros Fotiou, Nikolaos Loukas, Aimilia Mantzou, Vasileiοs Pergialiotis, George Valsamakis, Nikolaos Machairiotis, Sofoklis Stavros, Periklis Panagopoulos, Panagiotis Vakas, Christina Kanaka-Gantenbein, Petros Drakakis, Nikolaos Vrachnis

**Affiliations:** 1 Department of Obstetrics and Gynecology, Tzaneio General Hospital, 18536 Athens, Greece; 2 Medical School, National and Kapodistrian University of Athens, 11527 Athens, Greece; dionisisvrachnis@gmail.com (D.V.); alexandrosfotiou92@gmail.com (A.F.); 3 First Department of Paediatrics, Medical School, National and Kapodistrian University of Athens, Aghia Sophia Children’s Hospital, 11527 Athens, Greecechriskan@med.uoa.gr (C.K.-G.); 4 First Department of Obstetrics and Gynecology, Medical School, National and Kapodistrian University of Athens, Alexandra Hospital, 11528 Athens, Greece; pergialiotis@hotmail.com; 5 Second Department of Obstetrics and Gynecology, Medical School, National and Kapodistrian University of Athens, Aretaieion Hospital, 11528 Athens, Greece; gedvalsamakis@yahoo.com (G.V.);; 6 Third Department of Obstetrics and Gynecology, Medical School, National and Kapodistrian University of Athens, Attikon Hospital, Rimini 1, 12462 Athens, Greecepaninosrafaela@yahoo.gr (P.P.);; 7 Vascular Biology, Molecular and Clinical Sciences Research Institute, St George’s University of London, London SW17, UK

**Keywords:** calprotectin, parathormone (PTH), amniotic fluid, amniocentesis, small for gestational age (SGA), appropriate for gestational age (AGA), large for gestational age (LGA), inflammation, oxidative stress, fetal growth restriction

## Abstract

**Background**: During the early stages of human fetal development, the fetal skeleton system is chiefly made up of cartilage, which is gradually replaced by bone. Fetal bone development is mainly regulated by the parathyroid hormone parathormone (PTH) and PTH-related protein, with specific calprotectin playing a substantial role in cell adhesion and chemotaxis while exhibiting antimicrobial activity during the inflammatory osteogenesis process. The aim of our study was to measure the levels of PTH and calprotectin in early second trimester amniotic fluid and to carry out a comparison between the levels observed among normal full-term pregnancies (control group) and those of the groups of embryos exhibiting impaired or enhanced growth. **Methods:** For the present prospective study, we collected amniotic fluid samples from pregnancies that underwent amniocentesis at 15 to 22 weeks of gestational age during the period 2021–2023. Subsequently, we followed up on all pregnancies closely until delivery. Having recorded fetal birthweights, we then divided the neonates into three groups: small for gestational age (SGA), appropriate for gestational age (AGA), and large for gestational age (LGA). **Results:** In total, 64 pregnancies, including 14 SGA, 10 LGA, and 40 AGA fetuses, were included in our study. Both substances were detected in early second trimester amniotic fluid in both groups. Concentrations of calprotectin differed significantly among the three groups (*p* = 0.033). AGA fetuses had a lower mean value of 4.195 (2.415–6.425) IU/mL, whereas LGA fetuses had a higher mean value of 6.055 (4.887–13.950) IU/mL, while SGA fetuses had a mean value of 5.475 (3.400–9.177) IU/mL. Further analysis revealed that only LGA fetuses had significantly higher calprotectin concentrations compared to AGA fetuses (*p* = 0.018). PTH concentration was similar between the groups, with LGA fetuses having a mean value of 13.18 (9.51–15.52) IU/mL, while SGA fetuses had a mean value of 14.18 (9.02–16.00) IU/mL, and AGA fetuses had similar concentrations of 13.35 (9.05–15.81) IU/mL. The differences in PTH concentration among the three groups were not statistically significant (*p* = 0.513). **Conclusions:** Calprotectin values in the amniotic fluid in the early second trimester were higher in LGA fetuses compared to those in the SGA and AGA categories. LGA fetuses can possibly be in a state of low-grade chronic inflammation due to excessive fat deposition, causing oxidative stress in LGA fetuses and, eventually, the release of calprotectin. Moreover, PTH concentrations in the amniotic fluid of early second trimester pregnancies were not found to be statistically correlated with fetal growth abnormalities in either LGA or SGA fetuses. However, the early time of collection and the small number of patients in our study should be taken into account.

## 1. Introduction

Human fetal development results from a complex process, including, among others, the nutrition supply provided by the mother through a properly developed and functional placenta. This process is also driven by the fetus’s genetically determined growth potential and is affected by several epigenetic factors [1,2]. However, the precise cellular and molecular pathways underlying normal fetal development remain to be further elucidated.

Maternal pathology, such as hypertensive disorders, gestational diabetes, infections, and fetal pathology relating to genetic abnormalities, malformations, infections, and placental pathology, notably placenta insufficiency, are highly likely to impair normal fetal growth [3,4]. Among these, placental insufficiency is the most common cause of major fetal complications, including severe fetal growth restriction [5,6]. Mild placental insufficiency leads to fetal circulation redistribution to the brain, heart, and adrenal glands; this compensatory mechanism is known as the brain-sparing effect [7]. When an insufficient placental supply becomes critical, every organ and tissue of the fetus is affected, including bone development [8]. We do not know to what extent calprotectin or parathyroid hormone (PTH) contributes to the trend towards restricted bone development. On the other hand, maternal pathology, such as gestational diabetes, could cause LGA (large for gestational age) fetuses. LGA fetuses are complicated by excessive adipose tissue, which may affect the levels of oxidative stress.

Impaired or excessive fetal growth increases perinatal morbidity and mortality [9,10,11]. A fetus whose estimated fetal weight is lower than the third centile is classified as severe SGA (small for gestational age). The lower the fetal centile, the more increased the risk is for adverse pregnancy outcomes [12]. Similarly, in an LGA fetus, the perinatal complications increase as fetal macrosomia worsens. Importantly, early antenatal identification of fetuses with increased risk for SGA and LGA in the second and third trimesters enables the physician to more closely follow up on the case and planing of targeted interventions, if required, in order to enhance outcomes [13,14].

Previous studies have focused their research on various biomarkers in amniotic fluid and in the protein complex [15,16]. Amniotic fluid has a similar composition to that of fetal plasma and consists of various circulating biomarkers, which have an integral role in the establishment of a normal fetal environment [17]. Therefore, amniotic fluid shows itself to be an accessible tool for early recognition of any future deviation of normal fetal development, which manifests earlier at the cellular level or at the level of various mediators. A common link between SGA and preeclampsia is the presence of severe hypoxia, which, in turn, causes neutrophil activation [18,19]. Subsequently, the activated neutrophils release a number of hormones and mediators, including calprotectin: the latter composes up to 60% of the neutrophils’ soluble protein contained in the cytosol and expresses its action through cellular apoptosis [20].

During the early stages of fetal life, the embryonic skeleton is mainly composed of cartilage, which, as pregnancy progresses, is gradually replaced by bone. This process is necessary for the complex morphogenesis of fetal bones and is driven by a well-balanced equilibrium of bone absorption by osteoclasts and bone development by osteoblasts [21]. A fundamental component of normal fetal skeletal development is the active transport of minerals, in particular, calcium, phosphorus, and magnesium, through the placenta to achieve adequate mineralization and bone health [22,23]. Of note, human and animal data suggest that overall fetal bone development is regulated mainly by parathormone (PTH) and PTH-related protein [23].

Calprotectin is a complex of mammalian proteins S100A8 and S100A9, which is found inside the cytosol of neutrophil granulocytes. Moreover, calprotectin is also located, at lower concentrations, in monocytes and squamous epithelial cells [24]. During various inflammatory processes, the secretion of calprotectin has a substantial role in cell adhesion and chemotaxis and exhibits antimicrobial activity [25]. In today’s clinical practice, fecal calprotectin is used as a diagnostic test, with elevated levels pointing to ulcerative colitis or Crohn’s disease in children and adults [26].

As is known, based on several ultrasonographic studies, fetal bone development starts as early as the 11th gestational week. At this point of gestation, hematopoiesis also begins in the bone marrow, where, for the first time, neutrophil granulocytes are present in substantial numbers [27]. The exact underlying mechanism which coordinates the genesis of neutrophils in the bone marrow and, in consequence, the elevation of calprotectin concentrations is not yet fully elucidated [28]. However, future investigations should closely examine the simultaneous onset of bone development and hematopoiesis in order to shed light on the aforementioned process involving the increase in calprotectin levels.

Our study investigated for the first time, to our knowledge, whether PTH and calprotectin are present in the amniotic fluid from the early second trimester of pregnancy and, additionally, whether there is any correlation between these two mediators and fetal growth disorders such as SGA or LGA compared to AGA (appropriate for gestational age) fetuses.

## 2. Materials and Methods

In our prospective observational cohort study, amniotic fluid samples were collected from pregnant women who progressively enrolled from 2020 until 2022 based on the inclusion criteria. Specifically, the inclusion criteria were indications for invasive prenatal testing, such as an abnormal combined risk for chromosomal abnormalities in the first-trimester screening, increased nuchal translucency, advanced maternal age, or maternal request. After providing informed consent, all cases underwent amniocentesis in the early second trimester from 15 to 22 weeks. Multiple pregnancies, fetuses with congenital malformations, or pregnancies which showed abnormal karyotype results were excluded from the study. After collection, the amniotic fluid samples were centrifuged, and the supernatant was stored in Eppendorf tubes and kept at −80 °C. All pregnancies included were closely followed up until delivery.

During the time period between 15 and 20 weeks, when amniocentesis is performed, the fetal organs have completed their embryological development, with the exception of the fetal brain. However, different organs may reach their full functional capacity at earlier or later stages of pregnancy, which is being investigated in the current study.

Fetal birthweights were then recorded, after which the cases were divided into three groups. Neonatal birthweight assessment was made using gestational age-related fetal weight software which allocated the exact weight centile to each fetus [18]. A newborn with a birthweight less than the 10th percentile for a specific gestational age was characterized as SGA. On the other hand, if the birthweight exceeded the 90th percentile for a given gestational age, then the fetus was classified as LGA. All fetuses whose birthweight was between the 10th and 90th percentile for a specific gestational age were classified as AGA.

All the possible confounding factors were taken into account and were documented, such as maternal characteristics (e.g., age, weight, height, and parity), maternal habits (e.g., smoking and alcohol consumption), and gestational age at labor.

PTH concentrations in the amniotic fluid samples were determined using the electrochemiluminescence immunoassay “ECLIA” (Elecsys PTH immunoassay kit, Roche, Mannheim, Germany, Detection Range: 1.2–5.000 pg/mL, sensitivity: 1.2 pg/mL) on a Cobas immunoassay analyzer (Cobas e411). Calprotectin concentrations were determined using an ELISA kit (S-1011, BMA Biomedicals AG, Augst, Switzerland; Detection Range: 0–220 ng/mL, sensitivity: 0.686 ng/mL) [25]. Because the calprotectin concentrations in the amniotic fluid were found to be much higher than the detection range of the kit, serial dilutions of the samples were performed. All amniotic fluid samples required a 100-fold (1/100) dilution using the buffer solution of the calprotectin kit.

Informed consent was obtained from all women who participated in the study. The study was approved by the Ethical Committee for Research of Aretaieion University Hospital, Athens, Greece (319/26032021).

We assessed the results with the SPSS statistical program (IBM Corp., Armonk, NY, USA; Released 2012. IBM SPSS Statistics for Windows, Version 21) using parametric and non-parametric methods. The distribution of sample values was evaluated with the Kolmogorov–Smirnov test. In order to compare the PTH and calprotectin values between the three study groups (AGA, SGA, and LGA), we used the Kruskal–Wallis test. Variables that were not normally distributed are presented as median and interquartile ranges. Further post hoc pairwise analysis was carried out using Dunn’s test with Bonferroni correction. We set the level of significance at a *p*-value of less than 0.05. A Spearman’s rank correlation coefficient between PTH and calprotectin concentrations with all other arithmetic parameters was performed.

## 3. Results

In total, 64 pregnancies were included in our study, which were subgrouped based on their fetuses’ birthweights. More specifically, 14 fetuses were documented as SGA, 10 as LGA, and 40 as AGA. Table 1 summarizes the demographic characteristics of the cases composing the three groups. There was an anticipated difference in birthweight and birth centile as the latter was the basis according to which the different groups were determined. Thus, as expected, neonates in the LGA group weighed more than those in the AGA group and, more markedly, than those in the SGA group. Fetal sex and mode of delivery did not differ significantly among the groups.

**Figure 1:**
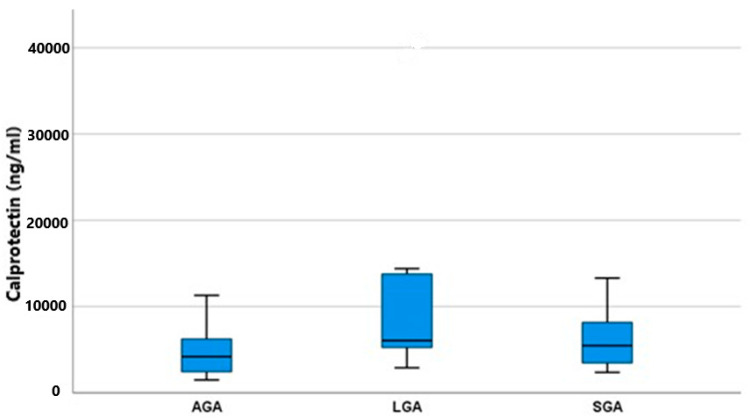
Calprotectin mean values (ng/mL) and IQR (Q1–Q2) among groups (AGA, SGA, and LGA).

Both substances, calprotectin and PTH, were detected in all amniotic fluid specimens, thus confirming their production. As shown in Figure 1, the concentrations of calprotectin differed significantly among the two groups, AGA and LGA (*p* = 0.033). AGA fetuses had a lower mean value of 4.195 (2.415–6.425) IU/mL, whereas LGA fetuses had a higher mean value of 6.055 (4.887–13.950) IU/mL. SGA fetuses had an in-between mean value of 5.475 (3.400–9.177) IU/mL. As shown in Table 2, the post hoc analysis among all possible pairs of our groups to determine the exact relationship between calprotectin and fetal growth revealed that only LGA fetuses had significantly higher calprotectin concentrations than AGA fetuses (*p* = 0.018). SGA fetuses, having a concentration between the two other groups, failed to reach statistical significance (SGA vs. AGA *p* = 0.103, SGA vs. LGA *p* = 0.421). It was evident that both fetal growth disturbances (overgrowth and growth restriction) induce calprotectin production, but this is augmented and overt in the case of fetal macrosomia.

**Figure 2:**
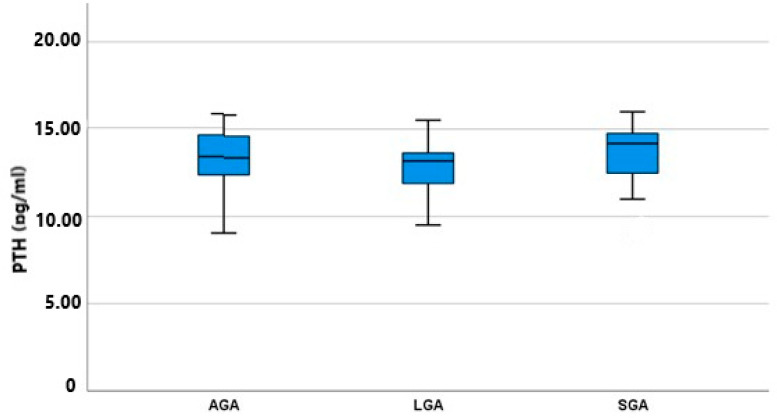
PTH mean values (pg/mL) and IQR (Q1–Q2) among groups (AGA, SGA, and LGA).

As shown in Figure 2, the amniotic fluid PTH concentration of the three subgroups did not differ significantly, with LGA fetuses having a lower mean value of 13.18 (9.51–15.52) IU/mL and SGA fetuses having a higher mean value of 14.18 (9.02–16.00) IU/mL. AGA fetuses had nearly the same concentration as SGA fetuses, measured at 13.35 (9.05–15.81) IU/mL. These differences among the groups were not statistically significant (*p* = 0.513); however, an inverse trend between fetal birthweight and PTH concentration was revealed. As shown in Table 3, we conducted a post hoc analysis among all possible pairs of our groups to identify any differences between specific groups masked under the simultaneous three-group analysis: this also failed to show any significant difference (SGA vs. AGA *p* = 0.427, SGA vs. LGA *p* = 0.249, and AGA vs. LGA *p* = 0.510).

Table 4 presents Spearman’s rank correlation between calprotectin, PTH concentrations, and all other parameters, such as age, weight, height, gestational age, birthweight, and percentile. No significant statistical correlation was found between these parameters other than a statistically significant inverse correlation between calprotectin and PTH.

## 4. Discussion

While prenatal medicine has made significant strides in the last few years, fetal growth abnormalities persist as one of the leading contributors to maternal and fetal mortality and morbidity. The underlying etiology is still unclear, necessitating further investigation. Our study sought to investigate the possible role of PTH and calprotectin in these pathological conditions through the evaluation of amniotic fluid concentrations of these biomarkers in early second trimester pregnancies. Our results showed that LGA fetuses’ calprotectin levels were statistically higher compared to AGA fetuses’ concentrations, while no statistically significant differences were observed between LGA–SGA and SGA–AGA. Furthermore, Spearman’s rank analysis revealed a statistically significant inverse correlation between calprotectin levels and PTH levels.

Since the results of our study did not reveal any statistical difference in calprotectin values between SGA and AGA, fetal calprotectin levels do not reflect the increased neutrophil activation and the excessive apoptosis suspected in SGA fetuses. An explanation for the lack of significant differences between the two groups could be either a low level of activated fetal neutrophils or a low level of calprotectin in their cytosol [29]. Another possible explanation is that fetal growth restriction, especially in severe forms, is characterized by hypoxia and inflammatory reactions [30], although these normally take place during the late-second or third trimester. This could possibly explain why we did not detect these changes in our study, our samples being collected during the early second trimester of pregnancy. Since calprotectin has proved to be a reliable biomarker of neutrophil activation, it may serve as a valuable prognostic marker for preeclampsia and fetal growth restriction (FGR) due to the associated maternal inflammatory response [31,32,33]. Past studies have reported possibly elevated maternal calprotectin in preeclampsia compared to controls, but this finding was not validated in other studies [34,35]. Nevertheless, the upregulation of calprotectin levels with advancing gestation in the SGA group could mirror an excessive inflammatory response.

According to the findings of our study, there is a statistically significant difference in calprotectin levels in LGA fetuses compared with AGA. This can be explained by the fact that overweight fetuses are in a state of chronic adipose tissue inflammation [36,37,38]. Excessive fat accumulation in adipose tissue is related to several types of oxidative stress and is responsible for the release of proinflammatory cytokines. The large excess of oxidants produced during oxidative stress marks the beginning of dysmetabolic syndrome, which develops beyond the range of obesity. One result of this disparity between oxidative and antioxidative processes is inflammation, which causes tissue damage, affects cellular equilibrium, and generates the development of complex metabolic disturbances [39,40,41]. Our confirmation that overweight LGA fetuses can possibly be in a state of low-grade chronic inflammation is consistent with the results of previous studies among obese children [37,42,43]. It is quite possible that LGA is a risk factor for obesity later in life [44], while fetal calprotectin levels in the second trimester may be considered an early predictive biomarker for neonatal or third trimester fetal obesity.

Since PTH is a fundamental regulator of normal fetal bone development, we also investigated whether there is any difference in PTH amniotic fluid concentrations among the study groups, SGA, AGA, and LGA. Previous studies have shown that the fetal parathyroid glands and the placenta produce PTH from the first trimester of pregnancy [45]. Yet, no previous study has measured PTH values in the amniotic fluid of the second trimester and correlated them with birthweight. PTH interacts with the PTH1R receptor, which is located in the placenta and the fetal kidneys and bones. Despite its low concentration in fetal circulation, PTH plays an essential role in active calcium transport through the placenta, regulates fetal calcium homeostasis, and ensures adequate fetal skeletal mineralization [46]. In order to cope with the unique developmental demands of the fetal skeleton throughout pregnancy, fetal calcium and bone metabolism are significantly altered, the fetus requiring sufficient calcium via the placenta to respond to the needs of the speedily mineralizing skeleton [47]. In the face of various pregnancy constraints, bone biomarkers can be a potent alternative option for the evaluation of bone formation and resorption alterations while exposing at the cellular level the complex bone metabolism [48,49]. Animal data have shown that FGR is a risk factor for altered bone mass and density as well as the development later in life of osteoporosis [50].

Our findings support the notion that reduced fetal growth and overgrowth are not the consequence of abnormal PTH production, nor is bone mineralization affected in either of these conditions. SGA and LGA fetuses are mainly characterized by impaired or augmented glycogen and fat accumulation in the midsection of their body [51,52]. In contrast, the fetal skeletal system generally remains unaffected [53]. In line with our results are reports from past studies [54,55] in which, by measuring bone biomarkers, they concluded that bone metabolism remains unaltered in FGR fetuses. More specifically, 20 AGA and 20 asymmetric FGR full-term singleton fetuses were enrolled in the above study at the time of birth. However, statistical analysis revealed no significant differences among AGA and FGR fetuses in full-term pregnancies as regards bone-specific alkaline phosphatase (BALP), alkaline phosphatase (ALP), osteocalcin (OC), PTH, urine cross-linked N-telopeptide of type I collagen (NTx), calcium, and phosphorus levels [54,56]. Other studies suggested that bone formation is diminished in SGA fetuses, as demonstrated by lower osteocalcin in cord blood [57,58]. However, osteocalcin is not a well-established marker of bone formation during the perinatal period [59,60]. According to previous studies, SGA fetuses exhibited a greater reduction in body weight than in body length. Placental weight, and thus the placenta as a site of exchange, corresponds better as a prognostic tool for the estimation of fetal weight than other parameters, such as fetal length. On the other hand, skeletal mass is correlated with length [61]. Therefore, calcium supply is typically sufficient for the relatively unaffected skeletal mass of an SGA fetus [61].

Based on the results of our study, PTH as a potential biomarker of fetal bone metabolism did not show any statistical difference among SGA, AGA, and LGA term fetuses. Thus, fetal bone metabolism is not affected in the early second trimester in cases that will be classified later in pregnancy as impaired or excess fetal growth as compared to controls.

Finally, the statistical analysis of our results revealed an inverse correlation between calprotectin and PTH. Although the latter has also been demonstrated in previous studies of inflammatory bowel disease in adults, this is the first time it has been studied in pregnancy to the best of our knowledge. According to one study, there is an inverse correlation between PTH, bone markers, and calprotectin [62]. This may indicate the influence of inflammation on bone mineral alterations [63]. Patients with inflammatory bowel disease, mainly those with Crohn’s disease, are at high risk for bone mineral modifications due to augmented cytokine release [64].

To our knowledge, the current study is the first to correlate calprotectin and PTH values in the amniotic fluid of fetuses during the early second trimester of pregnancy with actual fetal birthweight among SGA, AGA, and LGA fetuses. Furthermore, the inverse correlation of calprotectin and PTH in the amniotic fluid of fetuses in the early second trimester of pregnancy is also reported for the first time. A strength of our study is its prospective design, which reduces any selection bias and adds greater accuracy to our results.

The main limitation of our study is the relatively small sample sizes among all the study groups (SGA, AGA, and LGA). Larger studies need to be conducted in order to confirm our results and further elucidate the possible role of PTH and calprotectin in fetal growth disturbances and their correlation with oxidative stress. It is also of value in that it reveals some of the secrets of the as-yet little-known pathophysiological mechanisms of fetal growth disorders.

## 5. Conclusions

Our research has shown that calprotectin concentrations in the amniotic fluid in the early second trimester are higher in LGA fetuses compared to AGA and SGA. LGA fetuses are in a state of low-grade chronic inflammation due to excessive fat deposition, leading to oxidative stress and the release of various cytokines, such as calprotectin. On the other hand, we did not find any difference in the calprotectin values between SGA and AGA fetuses. This is either because of a lower degree of activated neutrophils or due to a still low calprotectin concentration in fetal neutrophils.

Our study revealed no difference in the PTH values of the amniotic fluid in the early second trimester among the SGA, AGA, and LGA fetuses. Since PTH is vital for active calcium transport through the placenta and regulates fetal calcium homeostasis, we can conclude that the bone mineralization process remains unaffected in the early second trimester of pregnancy, irrespective of any fetal growth disorder that will develop in the second half of pregnancy. In SGA fetuses, skeletal mass remains unaltered as these fetuses present with birthweight and placental mass reduction. Thus, the calcium supply for SGA fetuses is sufficient for their relatively unaffected skeletal mass.

Finally, the measurement of biomarkers, such as calprotectin, in the amniotic fluid makes possible early diagnosis of excessive fetal growth and forms the basis for future interventions aimed at reducing adverse perinatal outcomes.

## Figures and Tables

**Table 1:** Characteristics between groups (SGA, LGA, and AGA) are presented as median values (Q1–Q3) or frequencies. Discrete variables were analyzed with the chi-square test using Fisher’s exact test, and continuous variables were analyzed with the Mann–Whitney non-parametric test.

	AGA = 40	LGA = 10	SGA = 14	*p*-Value
**Maternal age**	37 (18–48)	35 (26–30)	36 (29–43)	0.391
**Maternal weight (kg)**	60.5 (47–93)	60.5 (55–100)	59 (49–90)	0.730
**Maternal height (cm)**	163 (152–175)	167 (165–172)	165 (160–175)	0.065
**Parity (nulliparity)**	9/34	3/8	4/12	0.787
**Gestational age (days)**	272 (240–284)	267 (261–275)	274 (263–282)	0.052
**Smoking (Yes)**	5/40	1/10	3/14	0.655
**Alcohol (Yes)**	3/40	0/10	0/13	0.417
**Neonatal weight (gr)**	3225 (1865–3965)	3775 (3550–4200)	2690 (2200–2850)	<0.001
**Neonatal sex (female)**	15/40	1/10	9/14	0.065
**Mode of delivery (VD)**	16/40	6/10	8/14	0.307
**Percentile**	42 (12–88)	93 (91–99)	5.5 (1–8)	<0.001

**Table 2:** Post-hoc pairwise analysis among the three study groups using Dunn’s test with a Bonferroni correction revealed a statistically significant difference in calprotectin values between LGA vs. AGA (statistical significance *p* < 0.05). The unadjusted values are presented in brackets.

Calprotectin	SGA	AGA	LGA
SGA	-	*p* = 0.103 (0.310)	*p* = 0.421 (0.805)
AGA	*p* = 0.103 (0.310)	-	*p* = 0.018 (0.048)
LGA	*p* = 0.421 (0.805)	*p* = 0.018 (0.048)	-

**Table 3:** Post hoc pairwise analysis among the three study groups using Dunn’s test with a Bonferroni correction revealed no statistical significance difference in PTH values (statistical significance *p* < 0.05). The unadjusted values are presented in brackets.

PTH	SGA	AGA	LGA
SGA	-	*p* = 0.427 (0.795)	*p* = 0.249 (0.748)
AGA	*p* = 0.427 (0.795)	-	*p* = 0.510 (0.659)
LGA	*p* = 0.249 (0.748)	*p* = 0.510 (0.659)	-

**Table 4:** Spearman’s rank correlation coefficient between calprotectin and PTH concentrations and other arithmetic parameters (**: correlation is significant at a level <0.001).

	Calprotectin	PTH	Age	Weight	Height	Gestational Age	Birthweight	Percentile
Calprotectin	1	−0.449 **	−0.015	0.078	0.061	−0.131	0.157	0.160
PTH		1	0.006	0.019	−0.148	0.054	−0.143	0.145

## Data Availability

The data presented in this study are available on request from the corresponding author. The dara are not publicly available due to general data protection regulation (GDPR).

## Abbreviations

SGA (small for gestational age), AGA (appropriate for gestational age), LGA (large for gestational age), PTH (parathormone), BALP (bone-specific alkaline phosphatase), ALP (alkaline phosphatase), and OC (osteocalcin).
